# Malignant upgrade in lesions of uncertain malignant potential in the breast (B3 lesions) – is open excision always necessary?

**DOI:** 10.1007/s10549-025-07632-7

**Published:** 2025-02-17

**Authors:** Felix Heindl, Janina Schiel, Carolin C. Hack, Niklas Amann, Sebastian M. Jud, Caroline I. Preuss, Lothar Häberle, Arndt Hartmann, Rüdiger Schulz-Wendtland, Matthias Wetzl, Matthias W. Beckmann, Ramona Erber

**Affiliations:** 1 Department of Gynecology and Obstetrics, Comprehensive Cancer Center Erlangen-EMN (CCC ER-EMN), Universitätsklinikum Erlangen, Friedrich-Alexander-Universität Erlangen-Nürnberg (FAU), Universitätsstraße 21–23, 91054 Erlangen, Germany; 2 Department of Gynecology and Obstetrics, Klinikum Mutterhaus Der Borromäerinnen, Trier, Germany; 3 Department of Gynaecology, Obstetrics and Gyn. Endocrinology, Johannes Kepler University Linz, Kepler University Hospital, Altenberger Strasse 69, 4040 Linz and Krankenhausstrasse 26-30, 4020 Linz, Austria; 4 Department of Gynecology and Obstetrics, Biostatistics Unit, Universitätsklinikum Erlangen, Friedrich-Alexander-Universität Erlangen-Nürnberg (FAU), Erlangen, Germany; 5 Institute of Pathology, Comprehensive Cancer Center Erlangen-EMN (CCC ER-EMN), Universitätsklinikum Erlangen, Friedrich-Alexander-Universität Erlangen-Nürnberg (FAU), Erlangen, Germany; 6 Institute of Radiology, Universitätsklinikum Erlangen, Erlangen, Germany; 7 Institute of Pathology, Universität Regensburg, Regensburg, Germany

**Keywords:** B3 lesions, Breast cancer, DCIS, Surgery

## Abstract

**Purpose:**

Unclear or suspicious breast findings are typically clarified by interventional breast biopsy. Lesions with uncertain malignant potential are grouped as B3 lesions in histopathology. The B3 group according to the European Working Group for Breast Screening Pathology (EWGBSP) comprises various breast lesions with different upgrade rates to invasive breast cancer (BC) or ductal carcinoma in situ (DCIS) if surgical removal is performed. The objective of this study was to investigate malignant upgrade rates to DCIS and/or invasive breast cancer (BC) after open surgical excision for the different B3 lesions.

**Methods:**

A total of 192 patients with histologically verified B3 lesions were followed up retrospectively for this analysis. Patients with the B3 lesions atypical ductal hyperplasia (ADH), flat epithelial atypia (FEA), and classical lobular neoplasia (LN1-2) were combined into one group, while cellular fibroepithelial lesions (CFL) and phyllodes tumors without suspicion of malignancy, as well as papillomas and radial scars/complex sclerosing lesions (RS/CSL) were summarized in two other groups. We investigated the association of the different B3 lesions with invasive BC or DCIS after open surgical excision.

**Results:**

Histopathological investigation revealed in 21 (10.9%) of the 192 patients invasive BC or DCIS after open surgical excision. The rate of patients with BC and/or DCIS significantly differed between the patient groups (p < 0.01, Fisher’s exact test): The highest rate was 17.5% (95% confidence interval (CI), 10.7–26.2) in patients within the group of ADH, FEA, and LN1-2. In the other two groups, fewer malignant lesions occurred. In the group with papillomas and RS/CSL the malignant upgrade rate was 4.3% (95% CI, 0.9–12.2), while within the group with CFL and phyllodes tumors without suspicion of malignancy no malignant upgrade was observed (0.0%, 95% CI, 0.0–16.9).

**Conclusions:**

B3 lesions harbor the risk of malignant upgrade after surgical excision. In our collective ADH, FEA, and LN1-2 had significant higher upgrade rates than other B3 lesions.

## Introduction

Core needle biopsies (CNB) and vacuum-assisted biopsies (VAB) are performed as standard of care to clarify unclear or suspected malignant breast lesions. Following histopathological examination of these diagnostic biopsies, they are categorized according to the B-classification system [1, 2]. For biopsies containing lesions of uncertain malignant potential, the category B3 is used. B3 lesions are diagnosed in approximately 5—10% of CNBs [3, 4]. Also due to the increasing use of mammography screening, an increasing detection rate of B3 lesions could be demonstrated [5–7].

B3 lesions represent a heterogeneous group of breast lesions with an increased risk of associated malignancy and show heterogeneity with the risk of an incomplete sampling [8, 9]. When B3 lesions detected via CNB or VAB undergo open excision, they are upgraded to malignancy, namely breast cancer (BC) or ductal carcinoma in situ (DCIS), in 9.9—35.1% of patients.

The most common representatives of B3 lesions are atypical ductal hyperplasia (ADH), classical lobular neoplasia (LN1-2), flat epithelial atypia (FEA), cellular fibroepithelial lesions (CFL) or phyllodes tumor without suspicion of malignancy, papilloma and other papillary lesions, and radial scar/complex sclerosing lesion (RS/CSL) [8–10]. LN1 and LN2 are former terms that now correspond to atypical lobular hyperplasia (ALH) and classic lobular carcinoma in situ (LCIS), respectively, in the 5th edition of the WHO tumor classification.

Considering the fact that not every B3 lesion carries the same malignancy risk, there is a tendency to further subdivide B3 lesions, enabling the derivation of clinical implications. ADH, FEA, and LN are considered lesions with the risk of associated DCIS or invasive BC. Lesions at risk of incomplete sampling include CFL or phyllodes tumors without suspicion of malignancy, intraductal papillomas, RS/CSL, hemangiomas, and atypical vascular lesions. Additionally, there are rare lesions within the B3 group, such as adenomyoepitheliomas, nipple adenomas, syringomatous tumors, microglandular adenosis, mucocele-like lesions, nodular fasciitis, desmoid-type fibromatosis, and uncertain spindle cell lesions, which, due to their rarity, are not further addressed in this work [10–14].

The clinical management of B3 lesions has undergone substantial evolution over time. Traditionally, every B3 lesion has been subject to open excision. However, recognizing that malignancy is not uniformly associated within B3 lesions, there has been a growing call to de-escalate treatment. To mitigate surgical over-treatment in patients with B3 lesions, there is a noticeable shift away from open surgical excision towards more interventional therapies, such as large volume VABs combined with regular monitoring or simply a follow-up examination [8, 9, 15].

Nevertheless, open excision is still suggested for certain types of B3 lesions due to the increased risk of DCIS or even invasive BC. Yet, it remains unclear which B3 lesions can safely forego open excision, and which should be recommended for it, considering the elevated risk of BC or DCIS. Hence, the objective of this study is to evaluate the incidence of BC or DCIS following open excision of B3 lesions in a German population.

## Methods

### Study population

**Fig. 1:**
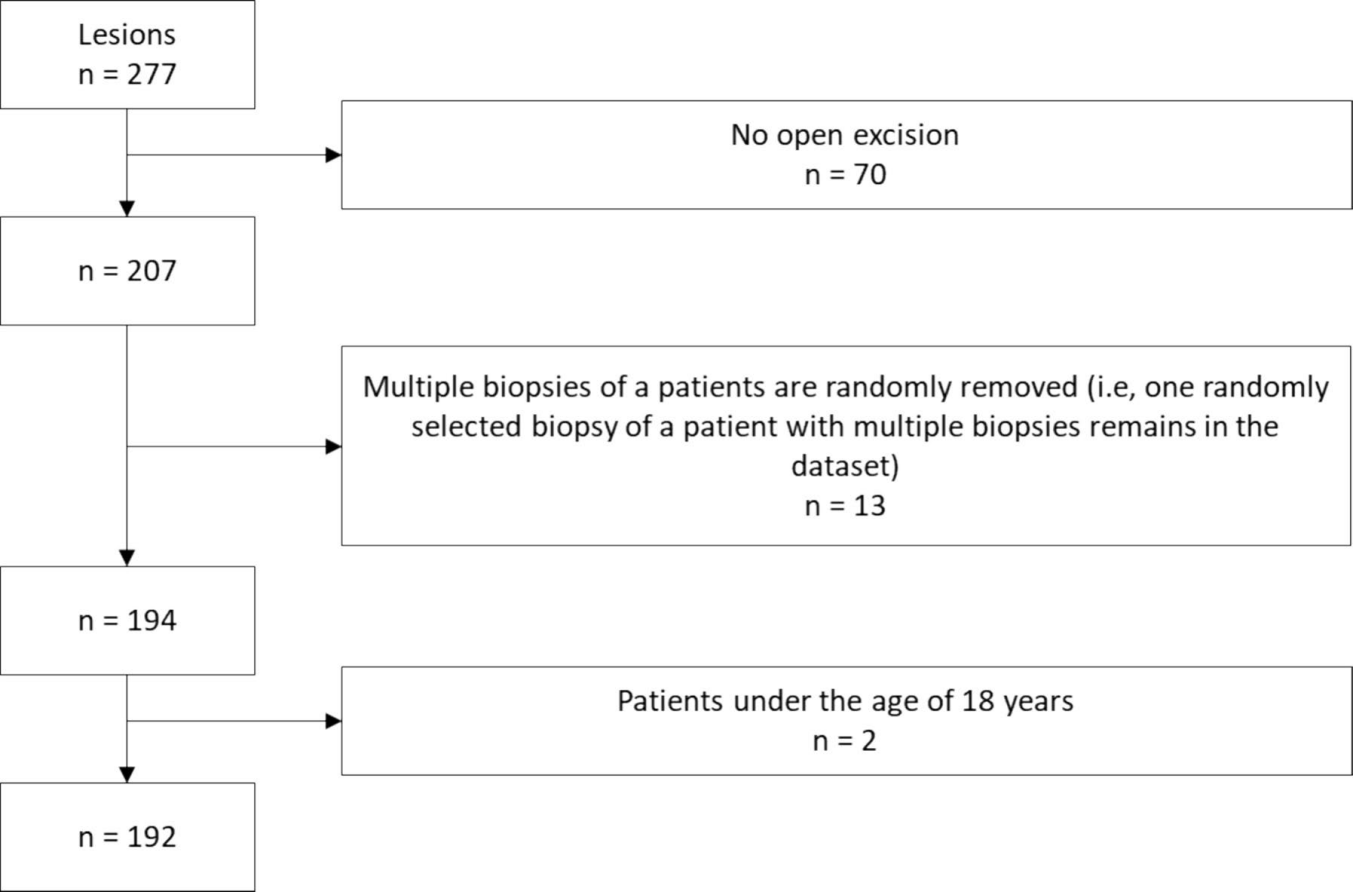
Flowchart of lesion selection

The cases included in this study were retrospectively selected from the database of the Institute of Pathology, Erlangen University Hospital, Erlangen, Germany. Every B3 lesion was counted as an individual lesion. For the period March 2013 until June 2019, a total of 277 histologically verified consecutive B3 lesions were identified in 251 patients who had undergone a CNB or VAB. Of these 277 B3 lesions, 70 lesions were not followed up by open excision (e.g., patients refused an open excision) or treated elsewhere. This resulted in 207 lesions in 194 patients. Additionally, multiple biopsies of a patient were randomly removed (i.e., one randomly selected biopsy of a patient with multiple biopsies remained in the dataset). Two patients younger than 18 years of age were also excluded. The final dataset for this study consisted of 192 patients – see also Fig. 1 for lesion selection.

### Histopathological workup of the B3 lesions

Prior to our retrospective study, the corresponding VAB or CNB tissue of each case was processed and evaluated according to the accredited diagnostics protocol of the Institute of Pathology, Erlangen University Hospital, Erlangen, Germany. This comprised formalin fixation, embedding into paraffin, at least six cutting levels and hematoxylin and eosin (H&E) staining. Slide evaluation was performed by board-certified pathologists experienced in breast pathology. Where appropriate, immunohistochemical stainings were used to confirm the H&E diagnosis. The report contained the B-classification according to current guidelines. Based on the biological relevance of the different B3 lesions, we have formed the following distinct groups for further analysis: ADH, FEA, and lobular carcinoma in situ, classical type and atypical lobular hyperplasia (LN1-2) are summarized in the group "Risk of associated invasive BC and DCIS”. The group “Fibroepithelial lesions” contained CFL lesions including phyllodes tumors without suspicion of malignancy. Potentially heterogeneous lesions with risk of incomplete sampling other than fibroepithelial lesion as Papillomas and RS/CSL were combined in the group “Papillomas/RS”. All lesions containing larger cellular atypia were grouped into “Risk of associated invasive BC and DCIS”.

According to the current 5th edition of the WHO tumor classification, non-invasive LN are divided into atypical lobular hyperplasia (ALH), classic lobular carcinoma in situ (LCIS), pleomorphic LCIS and florid LCIS. ALH and classic LCIS are synonymous with the former terms LN1 and LN2. Since the diagnoses were made before the current WHO classification of tumors, we used the terms LN1 and LN2 in this study.

### Surgery

All the open surgical excisions took place at the Department of Gynecology and Obstetrics, Erlangen University Hospital, Erlangen, Germany. Preoperatively, non-palpable lesions were marked by wire and palpable lesions by skin marking, respectively. If several lesions were present in one patient, these were removed simultaneously with unambiguous assignment. Open surgical excision was performed under general anesthesia. After open excision of the marked breast tissue, specimens were labeled for orientation. Consecutively, a specimen radiogram was obtained to confirm successful removal of the target lesion.

### Histopathological workup of the surgical specimens

After fixation of the specimens in formalin for at least 16 h, pathologic processing and reporting was performed according to current guidelines and the accredited in-house protocol, by board-certified pathologists of the Institute of Pathology, Erlangen University Hospital, Erlangen, Germany. In brief, margins were inked and the entire specimen was investigated. To confirm that the specimen was removed near the former biopsy area, special attention was given to the presence of a biopsy channel or a clip.

### Clinical data

Individual histological results from the open surgical excision were obtained for every B3 lesion from the database of the Institute of Pathology, Erlangen University Hospital, Erlangen, Germany. Basic epidemiological data were extracted from the patients’ records at the Department of Gynecology Erlangen. In cases of verified BC or DCIS during open excision, additional data, including tumor stage, grading, hormone receptor status, and HER2/neu status, were extracted from the patients' records.

### Statistical analysis

Patient and lesion characteristics are described using appropriate summary statistics. Mean and standard deviation are used calculated for continuous characteristics, frequency and percentage for categorical characteristics.

Primary study aim was to investigate whether the assessment of the lesion was associated with actual invasive BC or DCIS. For this purpose, the study population was divided into three groups according to the assessment of the lesion (i.e., "Risk of associated invasive BC and DCIS”, “Fibroepithelial lesions”, and “Papillomas/RS”), and for each group the rate of patients with resected tissue containing invasive BC or DCIS was calculated. These rates were compared using the χ2 test or Fisher’s exact test. The χ2 test was used if the expected number in each cell was greater than 5, Fisher’s exact test was used otherwise. Furthermore, we report malignant upgrade rates within the group “Risk of associated invasive BC and DCIS”. In the case that several different lesions of this specific group were detected in a single biopsy, the following assignment was made: ADH + FEA and/or LN1-2: ADH; FEA + LN1-2: LN1-2.

All of the tests were two-sided, and a P value of < 0.05 was regarded as statistically significant. Calculations were carried out using the R system for statistical computing, version 4.3.0, 2023.

## Results

### Patient characteristics

Between March 2013 and June 2019, a total of 192 patients received the diagnosis of a B3 lesion and underwent open excision of the lesion. In cases where a patient presented with multiple lesions, one lesion was randomly selected, resulting in the inclusion of only one lesion per patient in the final dataset. Patients and lesion characteristics are shown in Table 1.

**Table 1:** Patient and lesion characteristics

Characteristic		
Age (years)		50.0 (13.6)
BMI (kg/m^2^)		25.1 (5.6)
Patient from the mammography screening program	No	119 (62.0)
	Yes	54 (28.1)
	Unknown	19 (9.9)
Clinical findings leading to CNB or VAB	Palpation findings	64 (33.7)
	Microcalcifications	31 (16.3)
	Nipple secretion	8 (4.2)
	Abnormal findings on mammography	46 (24.2)
	Abnormal findings on breast ultrasound	20 (10.5)
	Mastodynia	14 (7.4)
	Miscellaneous	7 (3.7)
Inclusion criteria for genetic testing according to the German Consortium for Hereditary Breast and Ovarian Cancer	Not available	6 (3.1)
	Negative	163 (84.9)
	Positive	23 (12.0)
Side of the breast biopsy	Left	92 (47.9)
	Right	100 (52.1)
Type of breast biopsy	CNB	124 (64.6)
	VAB	68 (35.4)
Mammographic density	ACR a	4 (2.8)
	ACR b	82 (56.9)
	ACR c	40 (27.8)
	ACR d	18 (12.5)
BI-RADS	BI-RADS 0	2 (1.1)
	BI-RADS 2	3 (1.6)
	BI-RADS 3	2 (1.1)
	BI-RADS 4	172 (92.0)
	BI-RADS 5	8 (4.3)
ADH in breast biopsy	No	154 (80.2)
	Yes	38 (19.8)
FEA in breast biopsy	No	118 (61.5)
	Yes	74 (38.5)
LN1-2 in breast biopsy	no LN	179 (93.2)
	LN1	8 (4.2)
	LN2	5 (2.6)
Cellular fibroepithelial lesions in breast biopsy	No	159 (82.8)
	Yes	33 (17.2)
Phyllodes tumour without suspicion of malignancy in breast biopsy	No	177 (92.2)
	Yes	15 (7.8)
Papilloma in breast biopsy	No	123 (64.1)
	Yes	69 (35.9)
RS/CSL in breast biopsy	No	174 (90.6)
	Yes	18 (9.4)
Microcalcifications in breast biopsy	No	116 (60.4)
	Yes	76 (39.6)
Biopsy group: "Risk of associated invasive BC and DCIS”	No	89 (46.4)
	Yes	103 (53.6)
Biopsy group: “Fibroepithelial lesions”	No	172 (89.6)
	Yes	20 (10.4)
Biopsy group: “Papillomas/RS”	No	123 (64.1)
	Yes	69 (35.9)
Histological result from open surgical excision	Invasive BC only	1 (0.5)
	DCIS only	15 (7.8)
	Invasive BC and DCIS	5 (2.6)
	Benign	171 (89.1)

Patient and tumor characteristics, showing mean and standard deviation or frequency and percentage

*ACR* American college of radiology, *BI-RADS* Breast imaging reporting and data system

Mean patient age was 50.0 years (standard deviation (SD) 13.6), with patients most frequently having palpation findings (33.7%) prior to interventional biopsy. The mean time between interventional breast biopsy and surgical excision was 57.4 days (SD 34.0). 12.0% of the 192 patients met the inclusion criteria for genetic testing according to the German Consortium for Hereditary Breast and Ovarian Cancer [16, 17]. The most often used method for interventional breast biopsy was CNB, accounting for 124 cases (64.6%). With 52.1%, the majority of interventional biopsies, and thus the diagnosis of B3 lesion, were conducted in the right breast. In complementary mammographic diagnostics prior to breast biopsy, a BI-RADS 4 was most frequently observed, accounting for 92.0% of cases.

### Lesion characteristics

After interventional breast biopsy microcalcifications were detectable on specimen radiograms in 39.6%. In our dataset, the most frequently detected B3 lesion types were FEA (38.5%), and papillomas (35.9%), whereas lobular neoplasia were less common, comprising LN1 (4.2%) and LN2 (2.6%), respectively. The different B3 lesion types including frequencies are shown in Table 1.

The largest B3 subgroup was the “Risk of associated invasive BC and DCIS” group, comprising 103 cases of ADH, FEA, or LN1-2 (53.6%). In contrast, there were 20 cases (10.4%) in the “Fibroepithelial lesions” group and 69 patients (35.9%) in the “Papillomas/RS” group (Table 2).

**Table 2:** Histopathological results of the open excision

Assessment of the lesion	All patients (N = 192)	Patients with resected tissue containing invasive BC and/or DCIS (N = 21)	
	N	N	Rate with 95% CI in %
“Risk of associated invasive BC and DCIS”	103	18	17.5 (10.7, 26.2)
“Fibroepithelial lesions”	20	0	0.0 (0.0, 16.8)
“Papillomas/RS”	69	3	4.3 (0.9, 12.2)

*CI* Confidence interval

### Histological results from open surgical excision

**Fig. 2:**
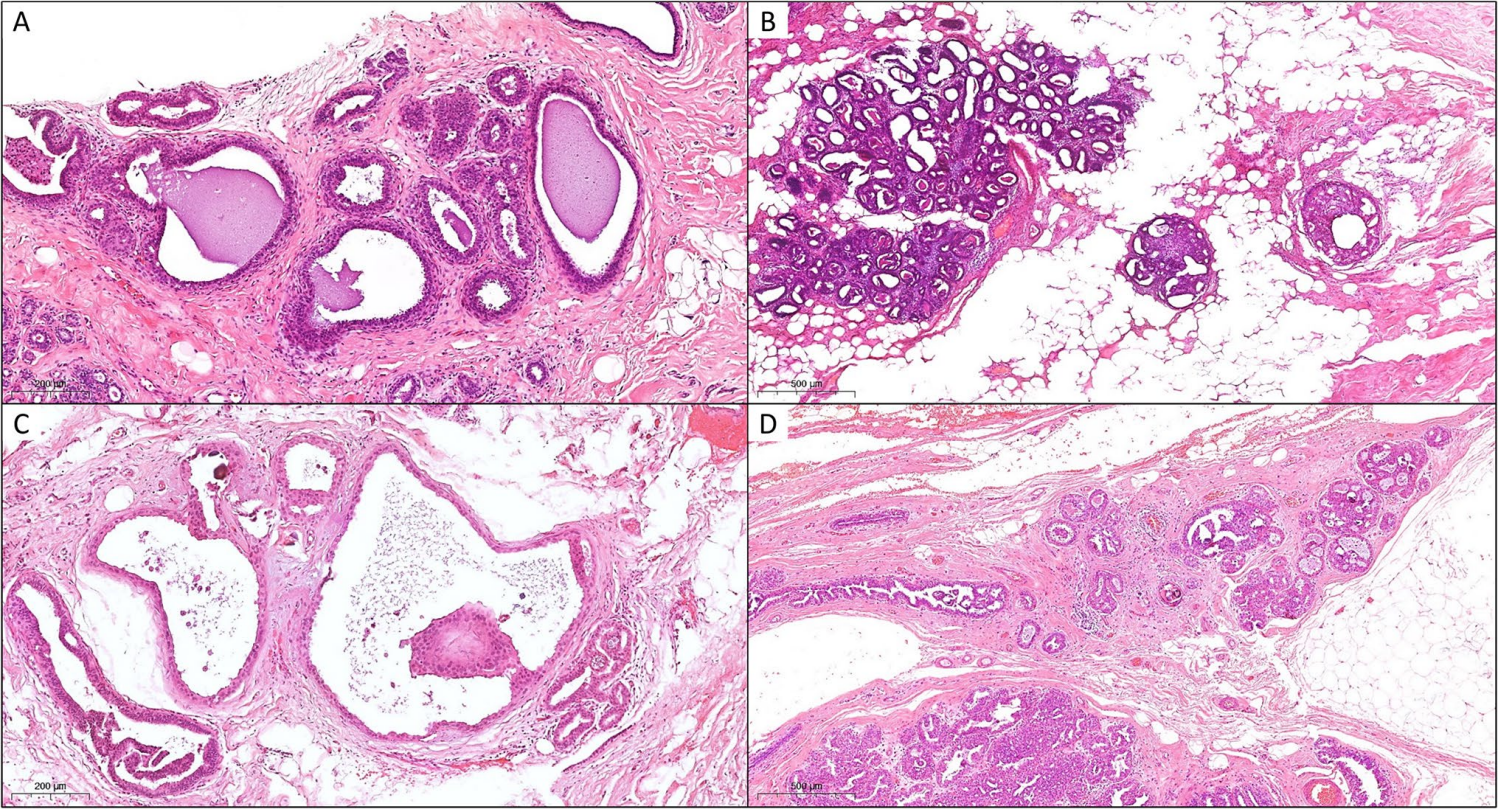
Examples for malignant upgrade from B3 to DCIS. Case 1 presented with **A** flat epithelial atypia (FEA) in the initial vacuum biopsy (H&E, 200x) but with **B** DCIS in the surgical specimen (H&E, 100x). Case 2 showed A) atypical ductal hyperplasia (ADH) in the initial vacuum biopsy (H&E, 200x) but with B) DCIS in the surgical specimen (H&E, 100x)

**Fig. 3:**
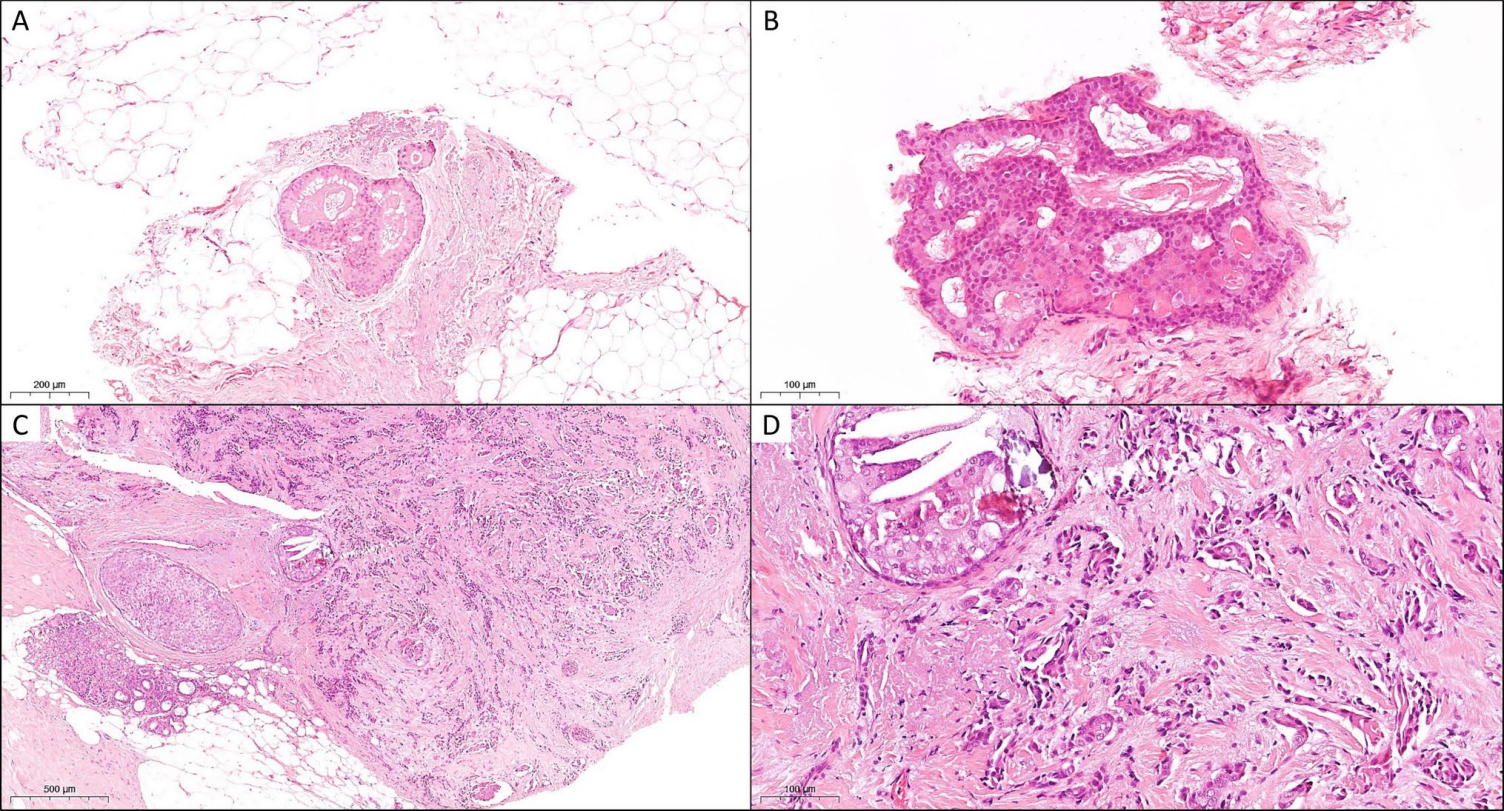
Example for malignant upgrade from B3 to invasive breast cancer and DCIS. In this case, the initial vacuum biopsy showed **A** flat epithelial atypia (FEA) (H&E, 200x) and **B** atypical ductal hyperplasia (ADH) (H&E, 400x). However, invasive breast cancer NST with associated DCIS was diagnosed in the surgical specimen (each H&E, **C** 100x, **D** 400x)

Overall, the resected tissue from 171 (89.1%) of the 192 patients contained benign tissue, whereas in 15 cases (7.8%) DCIS, in 5 cases (2.6%) BC and associated DCIS, and in one case (0.5%) BC were detected, respectively. Figure 2 provides examples for a malignant upgrade from B3 to DCIS, while Fig. 3 shows an example for malignant upgrade from a B3 lesion to invasive BC and DCIS. Table 3 and Table 4 provide detailed information on the histopathological findings, including prognostic as well as predictive parameters, for patients with a malignant upgrade to invasive BC or DCIS. In total we observed an upgrade rate of 10.9% (21 out of 192 cases) (Table 2).

**Table 3:** Upgrade cases to invasive Breast Cancer after open surgical excision

Case	Patient age	Histology of interventional biopsy	Breast Cancer type	Tumor size in mm	TNM Stage	Grading/Ki-67	ER / PR / HER2/neu
1	76	LN2	NST	11	pT1c pN0 cM0	G3/ 80%	10% / < 2% / negative
5*	54	ADH	NST	4	pT1a pNx cMx	G1/ < 5%	> 90% / 20% / negative
6*	60	ADH	ILC	27	pT2 pN2a cMx	G2 / 25%	100% / 5% / negative
18*	59	LN1	NST	3	pT1a pN0 cM0	G2 / 5%	> 80% / > 80% / negative
20*	75	ADH	NST	6	pT1b pN0 cM0	G2 / 2%	80% / 0% / negative
21*	72	LN2	ILC	55	pT3 pN0 cM0	G2 / 10%	100% / 15% / negative

*NST* No special type, *ILC* Invasive lobular carcinoma, *ER* Estrogen receptor, *PR* Progesterone receptor, *HER2/neu* Human epidermal growth factor receptor 2, *: indicating patients with concomitant invasive Breast Cancer and DCIS

**Table 4:** Upgrade cases to DCIS after open surgical excision

Case	Patient age	Histology of interventional biopsy	DCIS grade	DCIS size in mm
2	59	Papilloma	Intermediate-grade DCIS	4
3	45	Papilloma	Intermediate-grade DCIS	4
4	48	ADH	Intermediate-grade DCIS	3
5*	54	ADH	Low-grade DCIS	3
6*	60	ADH	Intermediate-grade DCIS	7
7	39	FEA	Low-grade DCIS	4
8	65	ADH	Intermediate-grade DCIS	3
9	52	ADH	Low-grade DCIS	9
10	67	ADH	Intermediate-grade DCIS	7
11	40	FEA	Low-grade DCIS	4
12	59	LN1	Low-grade DCIS	4
13	61	Papilloma	Intermediate-grade DCIS	1
14	45	FEA	Intermediate-grade DCIS	2
15	49	LN1	Intermediate-grade DCIS	13
16	38	LN1	High-grade DCIS	70
17	53	ADH	Intermediate-grade DCIS	12
18*	59	LN1	Intermediate-grade DCIS	1
19	48	FEA	Intermediate-grade DCIS	2
20*	75	ADH	Intermediate-grade DCIS	1
21*	72	LN2	Intermediate-grade DCIS	20

*DCIS* Ductal carcinoma in situ, *: indicating patients with concomitant invasive breast cancer and DCIS

The rate of patients with diagnosis of breast carcinoma or DCIS significantly differed between the patient groups (P < 0.01, Fisher’s exact test): The highest rate was 17.5% (95% confidence interval (CI), 10.7–26.2) in patients within the group “Risk of associated invasive BC and DCIS”. In the other two groups, fewer malignant lesions occurred. Among the 69 cases in the “Papillomas/RS” group, 3 DCIS were identified (4.3%, 95% CI, 0.9–12.2). In the "Fibroepithelial lesions" group, not a single invasive BC or DCIS was found (0.0%, 95% CI, 0.0–16.9) (Table 2). In patients within the group “Risk of associated invasive BC and DCIS” (n = 103 cases), 38 patients had a ADH lesion, 56 patients had a FEA lesion and 9 patients had a LN1-2 lesion. The rates of patients with resected tissue containing breast carcinoma or DCIS were 21.1% (8 of 38) for ADH, 7.1% (4 of 56) for FEA, and 66.7% (6 of 9) for LN1-2.

## Discussion

B3 lesions represent a heterogeneous group of breast lesions diagnosed by interventional biopsy as CNB or VAB. Due to their uncertain malignant potential, there is a lack of conclusive recommendations after diagnosis of B3 lesions. Since different B3 lesions can be present in one interventional biopsy, we decided to group the different lesions. Therefore, we subsumed biopsies that had various B3 lesions containing cellular atypia as ADH, FEA, and LN1-2 were named as “Risk of associated invasive BC and DCIS”. CFL lesions were grouped as “Fibroepithelial lesions”, while papillomas and RS/CSL were combined as “Papillomas/RS”. We hypothesized, that the group “Risk of associated invasive BC and DCIS” had the highest malignant upgrade rate.

This study of 192 patients investigated the malignant upgrade rate of B3 lesions after open surgical excision in a German population. The highest rate of invasive BC and/or DCIS was observed in the group “Risk of associated invasive BC and DCIS” containing the B3 lesions FEA, ADH, and LN1-2, respectively, with 17.5%. By contrast, the upgrade rates in the "Fibroepithelial lesions" group were 0%, and for "Papillomas/RS", it was 4.3%. Overall, we found an upgrade rate of 10.9%.

The group “Risk of associated invasive BC and DCIS”—containing ADH, FEA, and LN1-2—with 103 lesions represented the largest subgroup (53.6%) in our database. Compared to the two groups “Fibroepithelial lesions” and “Papillomas/RS”, we found a significant higher malignant upgrade rate of 17.5% (95% CI 10.7–26.2%).

For the B3 lesions ADH, FEA, and LN1-2, malignant upgrade rates of 28%, 11%, and 17%, respectively, were reported in a recent meta-analysis [18]. However, for the different lesions a large range of upgrade rates are published. In another review and meta-analysis of 93 studies, a comparable upgrade rate of 29% (95% CI 26–32%) for ADH alone after surgical excision is reported. Nevertheless, upgrade rates differed also for the distinct biopsy methods, with pooled upgrade rates of 42% (95% CI 31–53%), 23% (95% CI 19–27%), 32% (95% CI 22–43%) for ultrasound guidance, stereotactic guidance, or MRI guidance, respectively [19]. FEA shows a malignant upgrade rate in other meta-analyses between 5% (95% CI 3–6%), 8.8%, and 11.1%, respectively [20–22]. Identically, malignant upgrade in LN1-2 is published between 17% (95% CI 13–21%) in a recent meta-analysis and 3.7—28% in smaller study populations [18, 23–25]. Our combined malignant upgrade rate of 17.5% (95% CI 10.7–26.2%) for ADH, FEA, and LN1-2 fits into the wide range of different upgrade rates with various different study populations, types of breast biopsies, and other numerous preconditions. In contrast to the prementioned meta-analysis, we report lower malignant upgrade rates for ADH (21.1%) and FEA (7.1%), but a higher upgrade rate for LN1-2 (66.7%) [18].

The second largest group in our data are papillomas and RS/CSL with 69 out of 192 lesions (35.9%). For this group, we report 4.3% malignant upgrade. A limitation of our work is that our data lack information regarding the presence or absence of cellular atypia in papillary lesions. Atypical papillary lesions having the highest risk of upgrading to malignant after surgical excision in a meta-analysis [26]. In another systematic review and meta-analysis, an upgrade rate of 12% (95% CI 10–15) for papillomas, while for RS/CSL, an upgrade rate of 8% (95% CI 6–11) was reported [18]. In contrast, we report an upgrade rate of merely 4.3% (95% CI 0.9–12.2). One explanation for our relatively low upgrade rate could be, that we excluded papillary lesions or RS/CSL containing larger cellular atypia from the group “Papillomas/RS” and put them into the group with ADH, FEA, and LN1-2, since cellular atypia is the clinically dominant lesion.

Only including papillary lesions without atypia the prementioned meta-analysis presents a upgrade rate of 7% (95% CI 4–10)[18]. In line with our results, others found also a lower proportion of malignancies of 3.4% in non-atypical papillary lesions after surgical excision [27].

B3 lesions with fibroepithelial lesions represent the smallest group in our dataset comprising 20 lesions (10.4%). In line with other studies, we found no upgrade of fibroepithelial lesions to invasive BC and DCIS [8]. However, the focus in B3 lesions containing fibroepithelial lesions is not to rule out primary BC but malignant phyllodes tumors. In a retrospective analysis of 51 B3 lesions diagnosed as phyllodes tumors, only five were malignant phyllodes tumors after open excision [28]. In our data set, we could not observe an upgrade to malignant phyllodes tumors in the 20 fibroepithelial lesions. Another review found an upgrade rate of less than 2% for fibroepithelial lesions [29]. In a cohort of 215 patients with fibroepithelial B3 lesions, the upgrade rate to borderline or malignant phyllodes tumors was 2%, while LCIS was found in 1% [30]. Therefore, guidelines do not generally recommend the excision of fibroepithelial lesions [2]. A more refined approach for deciding whether to proceed with open excision is to consider the growth rate of the fibroepithelial lesion and suspicion for phyllodes tumor [31, 32].

Missing data on previous breast biopsies or previous diagnosis of invasive BC or DCIS are possible limitations of our work. Since earlier breast malignancy and breast biopsies are risk factors for BC and DCIS, an impact on the previous work cannot be excluded [33, 34]. Furthermore, our data lack information on symptoms prior to interventional breast biopsy. The presented retrospective data originate from a tertiary referral center in a university hospital maybe influencing patient acquisition. Since our work includes data until June 2019, our data lack information regarding the latest classification update on the WHO Classification of Tumours—Breast Tumours—in particular the new classification of lobular neoplasia [35]. Furthermore, our data lack information regard 70 cases that were not followed-up by open surgical excision. Some patients for example refused an open excision or were treated elsewhere so we cannot report upgrade rates for these cases.

In brief, the advantage of this study is the large number of patients included. The case only study design with the absence of specific inclusion criteria (e.g., type of biopsy or lesion type), the large study population of 192 patients, and grouping of B3 lesions separates this study from others. Since we identify statistically significant differences between the three B3 lesion groups, our results suggest distinct therapeutic implications. As proposed by other research groups, a surgical excision can be avoided for lesions with an upgrade rate of less than 2%. This is extrapolated analogously to the BI-RADS category 3, where a possible diagnostic delay is not associated with a worse prognosis [36]. Therefore, we suggest open surgical excision should be recommended for ADH, FEA, LN1-2, papillomas, and RS/CSL. Fibroepithelial lesions without growth tendency and without diagnostic uncertainties do not need to be excised.

## Conclusion

Lesions of uncertain malignant potential in the breast (B3 lesions) comprise different histopathologic distinct breast lesions with a heterogenous risk of ductal carcinoma in situ (DCIS) or invasive breast cancer (BC). In our study, we found the highest malignant upgrade rates for B3 lesions containing ADH, FEA, and LN1-2 after open surgical excision of 17.5%. Other B3 lesions in our data set had an upgrade rate of less than 5%. Based on our data, we recommend open surgical of the B3 lesions ADH, FEA, and LN1-2. For the other investigated B3 lesions at least follow-up examinations should be recommended.

## Data Availability

Data that support the findings of this study are available from the corresponding author upon reasonable request.

## Abbreviations

ACR: American college of radiology
ADH: Atypical ductal hyperplasia
ALH: Atypical lobular hyperplasia
B3: Lesions of uncertain malignant potential in the breast
BC: Breast cancer
BI-RADS: Breast imaging reporting and data system
CFL: Cellular fibroepithelial lesions
CI: Confidence interval
CNB: Core needle biopsy
CSL: Complex sclerosing lesion
DCIS: Ductal carcinoma in situ
EWGBSP: European working group for breast screening pathology
FEA: Flat epithelial atypia
H&E: Hematoxylin and eosin
HER2/neu: Human epidermal growth factor receptor 2
ILC: Invasive lobular carcinoma
LCIS: Classic lobular carcinoma in situ
LN: Classical lobular neoplasia
NST: No special type
VAB: Vacuum-assisted biopsy
RS: Radial scar
SD: Standard deviation
